# Current and emerging concepts in biological and analytical variation applied in clinical practice

**DOI:** 10.1111/jvim.15929

**Published:** 2020-10-21

**Authors:** Bente Flatland, Randolph M. Baral, Kathleen P. Freeman

**Affiliations:** ^1^ Department of Biomedical and Diagnostic Sciences College of Veterinary Medicine, University of Tennessee Knoxville Tennessee USA; ^2^ Paddington Cat Hospital Paddington New South Wales Australia; ^3^ Syn Laboratories – Veterinary Pathology Group (VPG), Torrance‐Diamond Diagnostic Laboratories University of Exeter, The Innovation Centre Exeter UK

**Keywords:** critical difference, dispersion, homeostatic set point, individuality, individualized reference interval, reference change value

## Abstract

A single laboratory result actually represents a range of possible values, and a given laboratory result is impacted not just by the presence or absence of disease, but also by biological variation of the measurand in question and analytical variation of the equipment used to make the measurement. Biological variation refers to variability in measurand concentration or activity around a homeostatic set point. Knowledge of biological and analytical variation can be used to facilitate interpretation of patient clinicopathologic data and is particularly useful for interpreting serial patient data and data at or near reference limits or clinical decision thresholds. Understanding how biological and analytical variation impact laboratory results is of increasing importance, because veterinarians evaluate serial data from individual patients, interpret data from multiple testing sites, and use expert consensus guidelines that include decision thresholds for clinicopathologic data interpretation. The purpose of our report is to review current and emerging concepts in biological and analytical variation and discuss how biological and analytical variation data can be used to facilitate clinicopathologic data interpretation. Inclusion of veterinary clinical pathologists having expertise in laboratory quality management and biological variation on research teams and veterinary practice guideline development teams is recommended, to ensure that various considerations for clinicopathologic data interpretation are addressed.

**Abbreviations**
BV: biological variation
CD: critical difference
CV: coefficient of variation
CV_A_: analytical variability; analytical coefficient of variation
CV_G_: between‐individual biological variation
CV_I_: within‐individual biological variation
Dev: deviation
HSP: homeostatic set point
II: index of individuality
iRI: individualized reference interval
pRI: population‐based reference interval
QCM: quality control material
RCV: reference change value

## 1 INTRODUCTION

Biological variation (BV) refers to variability in measurand concentration or activity around a homeostatic set point (HSP). Variability is a result of innate physiological factors and may or may not exhibit daily, monthly, or seasonal biological rhythms.^1^ Fluctuation around HSPs is assumed to be random, and BV is represented mathematically by coefficients of variation (CV) calculated from BV study data.^2^

In addition to BV, clinical laboratory results are impacted by factors affecting specimen quality (preanalytical factors, such as patient preparation, sampling technique, and specimen handling), analytical performance of the test system, and postanalytical factors such as data management practices (eg, plausibility checks to prevent erroneous data reporting).^1^, ^2^ Also important in the postanalytical phase is soundness of the tools (eg, reference intervals, decision thresholds) used by clinicians to interpret patient data.^2^

Our collective impression is that there is lack of formalized understanding among veterinarians of the impact of BV and analytical variation on measured results and how concepts from BV and laboratory quality management can be leveraged to facilitate patient data interpretation. Such understanding is of increasing importance, because veterinarians evaluate serial data from individual patients, interpret data from multiple testing sites, and use expert consensus guidelines that include decision thresholds for clinicopathologic data interpretation. Our purposes are to: (1) review current and emerging concepts in biological and analytical variation and (2) discuss how biological and analytical variation impact and can be used to facilitate patient or research subject data interpretation. The intended audience is veterinarians interpreting clinicopathologic data, particularly those who often interpret serial data from individual animals (eg, internists, criticalists, researchers) and veterinarians writing guideline documents incorporating laboratory decision thresholds.

## 2 BIOLOGICAL VARIATION

### 2.1 Components

Biological variation components are determined using a particular study design (see below). Biological variation calculations express the amount of variation in the data, expressed as coefficient of variation (CV), occurring within individuals (CV_I_; I for individual), between individuals (CV_G_; G for group), and caused by analyzer measurement performance (CV_A_; A for analytical).

For a given measurand, CV_I_ (within‐individual BV) expresses variation around the means of individual subjects and represents fluctuations around each subject's HSP.^1^, ^3^ The CV_G_ (between‐subject BV) expresses variation caused by differences among subject means and represents differences among the HSPs of the different subjects. The CV_I_ calculated from studies across human populations worldwide is generally similar, regardless of sex or ethnicity.^4^, ^5^ It is assumed that CV_I_ also is consistent across sexes and breeds for animal populations.

The CV_A_ (analytical variation) helps distinguish physiological fluctuations from variations associated with analyzer imprecision; as calculated from BV data, CV_A_ expresses variation among replicate measurements of the same specimens. Replicate measurements per specimen may not be possible under some circumstances, limiting assessment of CV_A_. For example, for species with small circulating blood volumes, small sample volumes may preclude replicate measurements per time point. For labile measurands (eg, ammonium, blood gases, or selected hematology measurands), replicate measurements may induce additional variation, and other approaches for estimating CV_A_ may be needed (see below).^6^

### 2.2 Considerations for study design

Basic design of BV studies is to repeatedly sample representative group of clinically healthy individuals under defined conditions; rigorous results can be determined with relatively small numbers of subjects. Variation components are calculated using either nested analysis of variance (nANOVA) or restricted maximum likelihood (REML) statistical approaches.^3^ Statistical power of estimating CV_I_ and CV_G_ depends on the ratio of CV_A_:CV_I_, the number of study subjects, the number of specimens per subject, and the number of replicate measurements per specimen.^7^

An aspect of BV study design that has not received adequate emphasis is standardization of time intervals among sample collections during the study. First, sampling intervals should be consistent throughout the study. If sampling intervals are varied within a single study, this introduces an additional source of variation that impacts BV components and complicates assessment of innate homeostatic variation. Such an impact on BV components may affect utility of other parameters calculated from BV data. Second, the duration of sampling interval should be optimized. With short time intervals between sampling, auto‐correlation of results is likely, contributing to smaller CV_I_ and CV_A_. Separate studies performed over increasing, but standardized, time intervals among collections enable recognition of when CV_I_ and CV_A_ cease to increase with increasing sampling intervals. Such analysis helps define the optimal sampling interval for retesting of patients when monitoring disease progress, response to treatment, or both.^8^, ^9^, ^10^

Statistical power of a BV study (and thus size of the study population) is impacted by the ratio of CV_A_:CV_I_. As this ratio increases (ie, higher CV_A_ compared to CV_I_), the number of subjects required increases so as to maintain statistical power.^7^ Current veterinary BV guidelines recommend that initial studies of biologic variation enroll 10‐15 subjects collected weekly for at least 4 to 6 weeks.^3^ A minimum of 15 subjects collected weekly for 6 weeks helps ensure sufficient study power, should there be a need to eliminate data (eg, outliers, analytical problems) before undertaking data analysis. For further details concerning veterinary BV study design and reporting, readers are referred to published guidelines.^3^

### 2.3 Biological variation concepts facilitating patient data interpretation

A single patient laboratory result represents only 1 of a range of possible values determined by a specified statistical probability of occurrence based on BV and measurement performance of the analytical method being used. Because BV impacts distribution of data for a given measurand in a given animal population, BV also impacts clinical utility of population‐based reference intervals (pRI), which by convention reflect the central 95% of data from a reference sample population. Optimally, patient data (and particularly serial data for a single patient) are interpreted in light of both BV and method analytical performance. The key clinical application of BV data to date has been to assess the medical importance of differences among serial patient results, particularly when such results are within the pRI (ie, interpreted as “normal”). Additional applications of BV data include assessing utility of pRI, determining dispersion of possible results for a single patient measurement, and determining how many repeat measurements or specimens are needed to estimate “true” patient measurand concentration or activity with a stated statistical probability of occurrence. Each of these applications is discussed in detail below.

Applying BV components to facilitate patient clinicopathologic data interpretation involves calculating various quantities using formulae incorporating CV_I_, CV_G_, and CV_A_, or some combination of these as explained below. Given complexity of many of the formulae, these quantities would be most easily applied in routine clinical practice if veterinarians had access to tools facilitating their calculation. Two of us are involved with on‐going efforts to develop such calculators and other tools.

For clinical application of BV data, ideally the CV_A_ used in the formulae should not come from the BV study itself, but rather should be determined for the testing site's actual instrument. The CV_A_ calculated from repeatability studies, in which replicate measurements of pooled patient specimens are performed under prescribed conditions, are preferred. Historical quality control material (QCM) data, obtained as part of a laboratory's routine quality control procedures, are another potential source for calculating CV_A_, but a limitation is that the QCM matrix may differ from the patient sample matrix (eg, aqueous solution versus plasma or whole blood) and may not produce the same result as patient samples. Studies have documented that CV_A_ derived from patient samples may differ from CV_A_ obtained from commercial QCMs for a variety of measurands, which is presumably related to the impact of storage, innate specimen matrix, or both.^6^, ^11^, ^12^, ^13^

Use of QCM‐based CV_A_ is reasonable if the measurand involved is labile (eg, ammonium, blood gases) and replicate measurement of individual or pooled patient samples is impacted by preanalytical factors. Use of QCM‐based CV_A_ also is reasonable if specimen volumes are small (eg, because of the logistics of blood sampling in certain species), precluding replicate analysis, if sample matrix (eg, whole blood) precludes pooling of individual animal specimens, or some combination of these factors. If using historical control data to calculate CV_A_, the International Standards Organization standard 15189 recommends that estimates of CV_A_ be based on “intermediate precision conditions,” which generally is interpreted to mean several months' of data incorporating at least 100 control measurements.^14^ Several months' of data should be readily available in diagnostic laboratories measuring ≥1 QCM daily. There is precedent for basing CV_A_ calculations on smaller data sets (eg, n = 20, or even n = 5 QCM measurements), particularly in the in‐clinic setting, where logistical or financial constraints may be limiting.^13^, ^15^, ^16^ Although precedent exists for estimating CV_A_ on as few as n = 5 replicate measurements,^15^ we suggest at least n = 10 to 20 in any setting.

### 2.4 Index of individuality and limitations of population‐based reference intervals

Individuality of a measurand refers to the ratio of (CV_I_ + CV_A_) and CV_G_. In simplistic terms, high individuality means individuals' results for that measurand vary greatly (ie, variability among the HSPs of different individuals exceeds variability caused by fluctuation around individual set points and analytical variation combined). Low individuality means individuals are less unique (ie, variability between individual set points is less, even as fluctuation around an individual's own set point may be variable).

Individuality is represented mathematically by the index of individuality (II). Current veterinary recommendations for II calculation are to use the formula:

$$\mathrm{II}=\frac{{\mathrm{CV}}_{G}}{\sqrt{{\mathrm{CV}}_{I}^{2}+{\mathrm{CV}}_{A}^{2}}}.$$

With this formula, a numerically high II (>1.67) indicates high individuality, and a numerically low II (<0.7) indicates low individuality. An II between 0.7 and 1.67 indicates intermediate individuality.^3^ Earlier human and veterinary medical literature used the inverse of this formula, which is less intuitive because a numerically low II (<0.6) indicates high individuality, and numerically high II (>1.4) indicates low individuality.^17^, ^18^

The pRI reflects the distribution of results for a given measurand in a clinically healthy population. Mathematically, pRI represent the central 95% of data from a reference sample population, and a guideline for de novo generation of veterinary reference intervals is published.^19^ It is important to understand that upper and lower reference limits are each statistical estimates, the soundness of which depends on the underlying data (eg, size of the study population, analytical performance of the instrument or method, and handling of outliers during data analysis). Because of measurement bias among commonly used analytical methods and laboratory settings, the pRI is considered specific to individual instruments, methods, or both, unless formal statistical validation has confirmed applicability of the pRI to other settings.^19^ Utility (ie, diagnostic sensitivity and specificity) of any pRI for disease diagnosis depends on demographic similarities between the reference sample and patient population, on statistical soundness of the reference limit estimates, and on the mathematical relationship of CV_I_ and CV_G_ for the measurand in question. The last is judged using II.^18^, ^19^

**FIGURE 1:**
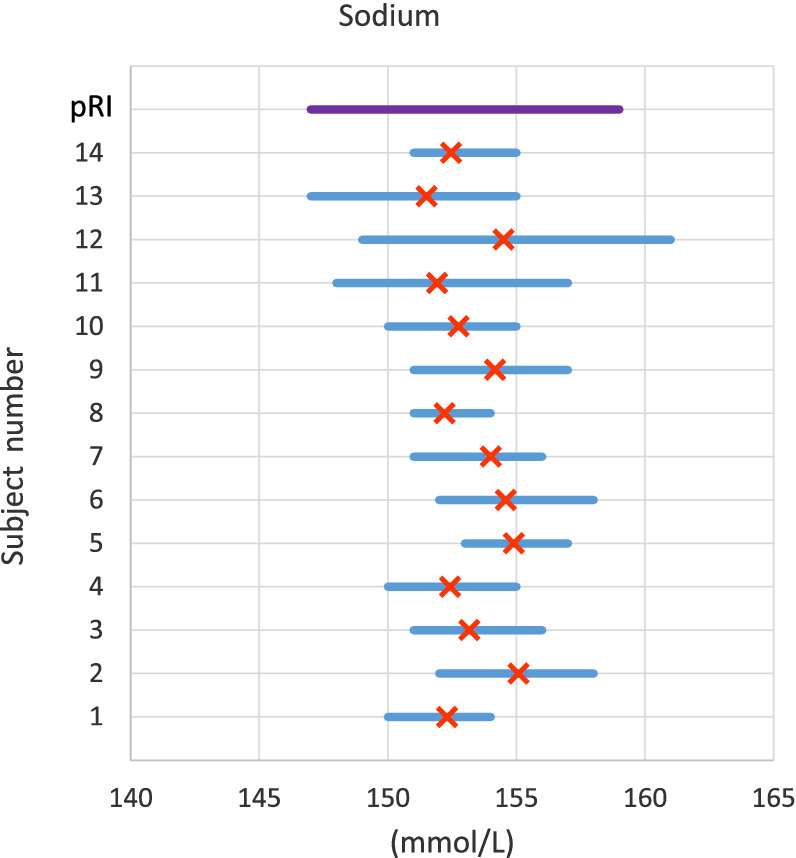
Demonstration of low individuality using plasma sodium concentration in cats. Data are from a prior published study and illustrate the variation and mean of results from 14 cats over 6 weeks.^41^ Measurand concentration is given on the x‐axis, and subjects are shown consecutively ascending the y‐axis. The purple line at the top of the graph, parallel to the x‐axis, represents pRI and is shown for perspective. Blue lines parallel to the x‐axis represent the range of values for each study subject, and orange crosses represent the mean for each subject. Because of low individuality of sodium, there is not much variability of the mean value for each subject (range of means, 151.5‐155 mmol/L). An iRI for each subject (not shown) would likely approximate the pRI, and pRI is expected to have good diagnostic sensitivity

**FIGURE 2:**
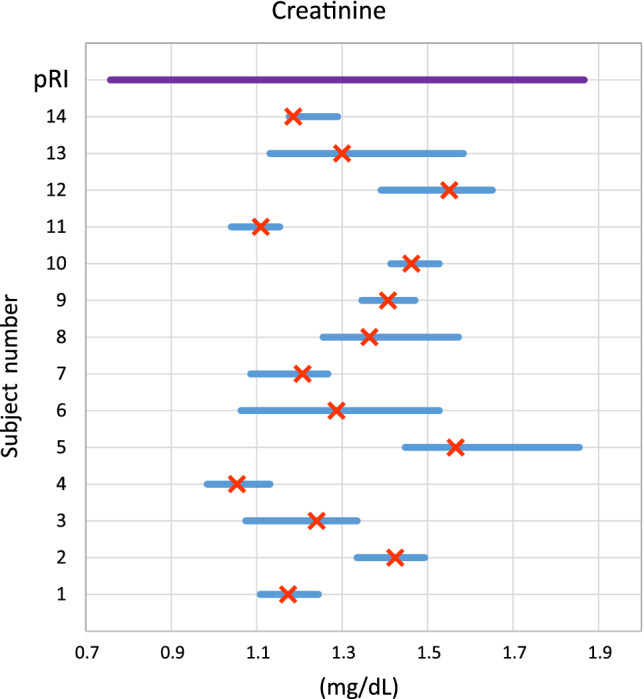
Demonstration of high individuality using plasma creatinine concentration in cats. Data are from a prior published study and illustrate the variation and mean of results from 14 cats over 6 weeks.^41^ Measurand concentration is given on the x‐axis, and subjects are given consecutively ascending the y‐axis. The purple line at the top of the graph, parallel to the x‐axis, represents pRI and is shown for perspective. Blue lines parallel to the x‐axis represent the range of values for each study subject, and orange crosses represent the mean for each subject. Due to high individuality of creatinine, there is variability of the mean values for each subject (range of means, 1.1‐2.0 mg/dL). iRI for a given individual subject (not shown) is expected to be different from the pRI, and pRI is expected to be less diagnostically sensitive

For measurands with high individuality (more marked variability among individual HSPs), pRIs have limited diagnostic sensitivity because data variation across the population is wider than variation within individuals comprising the population. For high‐individuality measurands, a given patient could have a medically important change in measurand concentration or activity (exceeding usual HSP fluctuation, suggesting disease) but the measured result still could fall within the pRI (ie, be interpreted as “normal”).^18^, ^20^ Population‐based reference intervals have greater diagnostic sensitivity for measurands with low individuality, because of lesser variation among individual HSP. Figures 1 and 2 illustrate these concepts graphically. Example calculations are provided in the Supplemental Appendix.

### 2.5 Homeostatic set point and critical number of samples

Many veterinary measurands are normally distributed when measured in a clinically healthy population. Given this fact, laboratory results from patients with disease can be interpreted in light of an estimated true patient concentration or activity based on health. This health‐based estimate for a given individual, measurand, and laboratory analyzer is simply the mean of repeat results from multiple specimens determined over time when the patient was known to be clinically healthy. This mean is known as the HSP.

Although a single laboratory result obtained during health can be used for estimation of HSP, a more accurate estimation of HSP is based on averaging multiple results (eg, from multiple annual wellness checks). The critical number of samples needed to estimate HSP with a given precision can be calculated based on SE of the mean estimate.^21^ For most measurands in dogs, cats and horses, HSP can be estimated with 95% probability from <4 samples (Table 1). In Table 1, the critical number of samples was calculated for each measurand and species according to the formula:

$$\text{Critical number}={\left(Z\times \frac{\sqrt{{\mathrm{CV}}_{I}^{2}+{\mathrm{CV}}_{A}^{2}}}{\mathrm{Dev}}\right)}^{2}$$

where Z is a Z factor from the standard normal distribution (= 1.96 for 2‐sided 95% probability), CV_A_ is analytical variation, CV_I_ is within‐individual BV, and Dev refers to allowable percentage deviation from the true HSP. Generally, 10% allowable deviation is chosen,^21^ which is appropriate for measurands with CV_I_ ≥ 6.67 but too high for low individuality measurands (eg, electrolytes). For measurands with CV_I_ < 6.67, we recommend Dev = 1.5 × CV_I_, up to a maximum Dev of 10%.

**TABLE 1:** Critical number of specimens for estimation of homeostatic set point for selected measurands in dogs, cats, and horses

Measurand	Species					
	Dog		Cat		Horse	
	95% probability	80% probability	95% probability	80% probability	95% probability	80% probability
Biochemistry						
Albumin	2	1	2	1	1	1
ALP	5	2	4	2	NA	NA
ALT	8	3	7	3	NA	NA
AST	4	2	9	4	4	1
Chloride	2	1	2	1	2	1
Cholesterol	5	2	2	1	NA	NA
Creatinine	6	3	4	2	3	1
Globulin	NA	NA	2	1	1	1
Glucose	3	1	3	1	3	1
Calcium	2	1	2	1	2	1
Magnesium	NA	NA	2	1	NA	NA
Phosphate	7	3	3	1	6	1
Potassium	2	1	2	1	4	1
Sodium	NA	NA	2	1	3	2
Total Protein	2	1	2	1	2	1
Triglycerides	50	21	4	2	4	1
Urea	2	1	4	2	4	1
SDMA	11	5	18	8	NA	NA
GGT	NA	NA	NA	NA	7	1
Hematology						
RBC	2	1	2	1	2	1
Hemoglobin	2	1	2	1	2	1
Hematocrit	2	1	2	1	2	1
MCV	1	1	2	1	1	1
MCH	2	1	2	1	1	1
MCHC	2	1	NA	NA	1	1
RDW‐CV	2	1	2	1	1	1
WBC	10	5	14	6	3	1
Neutrophils	26	11	25	11	6	2
Lymphocytes	5	2	25	11	11	3

*Notes*: The critical number of samples for each measurand and species below was calculated using Z factors of 1.96 (representing 95% bidirectional statistical probability) and 1.28 (representing 80% bidirectional statistical probability). Dev = 10% was used for those measurands with CV_I_ > 6.67%. For measurands with CV_I_ < 6.67%, Dev = 1.5 × CV_I_ was used. See formula in text. CV_I_ values used for each measurand and species are from the veterinary biological variation database website.^32^

Abbreviations: ALP, alkaline phosphatase; ALT, alanine aminotransferase; AST, aspartate aminotransferase; CI, confidence interval; GGT, gamma glutamyl transpeptidase; MCH, mean cell hemoglobin; MCHC, mean cell hemoglobin concentration; MCV, mean cell volume; NA, not available for this species; RBC, red blood cell; RDW‐CV, red blood cell distribution width (coefficient of variation); SDMA, symmetric dimethylarginine; WBC, white blood cell.

Wellness testing occasionally yields results outside the pRI. Careful consideration should be given to whether results outside pRI represent subclinical disease, or whether they reflect dispersion associated with BV or analytical variation.

### 2.6 Reference change value and individualized reference intervals

Reference change value (RCV), individualized reference intervals (iRI), and critical difference (CD) are calculated from BV data and can be used to facilitate patient data interpretation. Each is explained in detail below. Although most often calculated in the context of high individuality measurands (for which pRI has limited diagnostic sensitivity), these also can be calculated and used for measurands with low individuality.

For measurands with high individuality, calculation of RCV can facilitate understanding of whether observed changes in serial patient data are likely to be clinically relevant. The RCV is expressed in units of percent and calculated as:

$$\mathrm{RCV}=Z\times \sqrt{2\;}\times \sqrt{{\mathrm{CV}}_{I}^{2}+{\mathrm{CV}}_{A}^{2}}$$

where Z is a Z factor and the numeral 2 refers to evaluating 2 serial measurements. The Z factor reflects statistical probability (typically 90%, 95%, or 99%) of the RCV estimate and can be chosen to reflect uni‐ or bi‐directional change, as applicable.^3^ For many routine measurands, the chosen Z factor is 1.96, reflecting bidirectional change and 95% statistical probability. However, if clinical interest is only for decreased or only for increased results, then different Z factors with different probability can be used.

The RCV is used to assess whether the percentage difference between an HSP and a measured result is clinically relevant. The RCV formula must be adjusted if > 2 serial values are being interpreted.^20^ If the percentage change from HSP is numerically higher than the RCV, the change is interpreted as clinically relevant. If the percentage change from HSP is numerically less than the RCV, it cannot be excluded that change is caused by biological or analytical variation.^18^ Because it is expressed as a percentage, RCV, once calculated for a given measurand, instrument, and species, is applicable to patient results of variable concentrations or activities.

The RCV can be converted from a percentage to measurand units; expressed thus, it is known as CD. The RCV (in units of %) and CD (in measurand units) both represent the amount of change needed for a patient result to be considered clinically relevant. ^22^, ^23^ For example, the RCV for creatinine may be 30% in a particular species; if applied to a starting serum or plasma creatinine concentration of 1.0 mg/dL, the CD is 0.3 mg/dL. The CD obviously will vary according to the starting value from which it is calculated. Thus, a single CD value should not be applied broadly to a range of concentrations. If CD is applied to the HSP to create an interval, this is referred to as an iRI (sometimes referred to as subject‐based reference values).^18^, ^19^ Continuing the example above, an individual patient with a serum or plasma creatinine concentration HSP of 1.0 mg/dL would have an iRI of 1.0 ± 0.3 mg/dL, or 0.7 to 1.3 mg/dL. For any given measurand, HSP obviously will vary by individual. Thus, an iRI should not be applied broadly to multiple individuals, even of the same species or breed. Further example calculations are provided in the Supplemental Appendix.

In veterinary medicine, a major limitation of RCV and iRI use is that existing veterinary BV data are still being developed in scope and quality. Publication of a guideline for designing and reporting veterinary BV studies should help standardize future studies and improve data quality.^3^ Similar to pRI, another limitation of iRI is that these represent the central 95% of fluctuation around a patient's HSP, and thus outlying values close to the iRI limits may or may not be pathologic. A third limitation of RCV and iRI is that imprecision (CV_A_) of individual instruments used for assessment of veterinary patients may not be fully characterized, particularly in the in‐clinic setting.

A fourth potential limitation is that BV in health may or may not be the same as BV in disease. Studies in humans have shown that, for many common measurands in routine hematology and biochemistry, BV in health and in chronic stable disease are similar enough that applying RCV and iRI developed using BV data from healthy individuals is reasonable for patients with chronic, stable disease.^5^ This also appears true in animals, but limited studies have indicated differences for cardiac measurands for dogs with mitral disease.^24^, ^25^, ^26^ Less well characterized is similarity of BV data in health and acute disease. For measurands having a numerically smaller CV_I_ (and thus RCV) in health than in disease, use of RCV calculated from healthy subject data may cause false positive results (ie, a conclusion of clinically relevant change) in diseased patients.^27^, ^28^

Measurand concentrations in health are believed to fluctuate, similar to a sine wave, over time, and a fifth limitation of RCV is that the sampling interval of BV studies impacts CV_I_ calculated from the data. Because BV studies sample the same, relatively small group of individuals repeatedly over time, BV data are subject to the phenomenon known in statistics as autocorrelation.^29^ Autocorrelation (which can result in numerically smaller CV_I_ and therefore RCV) is more pronounced with samples collected over a short time period (eg, hourly or daily) than in those collected over a longer time period (eg, weekly).^27^ Impact of sampling intervals >1 week on BV study data is unknown. Theoretically, the sampling interval of BV data used to calculate RCV and iRI should be similar to the sampling interval of patient results to which RCV or iRI are applied. This is particularly true for short patient sampling intervals, as may occur in emergency and critical care settings. In such settings, sampling interval of serial patient data may be very short (hours or days). If RCV and iRI derived from a longer sampling interval (eg, weekly) are used to interpret such data, then CV_I_ (derived from the BV data) may be larger than actual variation occurring within the patient, risking a false negative interpretation of serial changes (ie, that change is not necessarily clinically relevant).^27^ On the other hand, studies of various analytes in humans indicate that CV_I_ increases as the sampling interval increases, up to 4 or 5 days, and then remains stable for longer sampling intervals, up to bimonthly samples obtained over a year or more.^8^, ^9^, ^10^ Although not formally studied, it seems likely that results from annual veterinary wellness testing could be assessed using BV data collected with weekly sampling intervals.

Finally, similar to the pRI model, the RCV model assumes Gaussian distribution of CV_I_ and CV_A_, when distribution may be skewed for some clinical measurands. This limitation can be mitigated by calculating RCV using a lognormal approach, a strategy most applicable to measurands having numerically large CV_I_ (>30%). However, measurands with such a large CV_I_ will have a high RCV and thus require large changes to be considered clinically relevant. In general, large changes in clinical pathology results are straightforward to interpret. In most circumstances, using the regular RCV model with Gaussian assumptions is appropriate.^28^

### 2.7 Dispersion

Given that an individual laboratory result represents 1 of a range of possible values determined by a specified statistical probability of occurrence based on CV_I_ and CV_A_, if CV_I_ and CV_A_ are known, this range of possible values, known as dispersion, can be estimated.^2^, ^30^, ^31^ Calculation of dispersion includes the number of specimens and number of replicate analyses used to determine an individual result. Although preanalytical factors impact measurement results, the following discussion of dispersion assumes that preanalytical factors are controlled. Mathematically, dispersion (*D*) is calculated as:

$$D=\pm Z\times \sqrt{\frac{{\mathrm{CV}}_{I}^{2}}{n_{s}}+\frac{{\mathrm{CV}}_{A}^{2}}{n_{a}}}$$

where Z is a Z factor, n_s_ is the number of patient samples measured, and n_a_ is the number of replicate measurements. Note that this is a reconfiguration of the SE of the mean calculation used to calculate critical number of samples, above. This configuration of the formula solves for *D*, and the number of specimens taken is known; whereas for critical number, the number of specimens is being calculated and *D* is represented by Dev, which is chosen to be ≤10%. If a single patient sample is measured once (the usual approach for clinical patient samples), the formula simplifies to^2^, ^30^, ^31^: $D=\pm Z\times \sqrt{{\mathrm{CV}}_{I}^{2}+{\mathrm{CV}}_{A}^{2}\;}$.

Calculating *D* and understanding the range of possible values represented by a single measured result can facilitate patient data interpretation, particularly for data at or close to reference limits or clinical decision thresholds. Understanding the dispersion associated with a particular reference interval limit or medical decision threshold (cutoff value) helps ensure that a measured result obtained for a given individual using a particular analyzer is correctly interpreted, and that clinical relevance is not attributed to a patient result that may only reflect biologic or analytical variation or both.

The concept of dispersion emphasizes the contribution of CV_A_ and CV_I_ to individual test results, and the relationship between these. If CV_A_ is substantially (eg, 10‐fold) less than CV_I_, averaging the results of the 2 samples decreases dispersion more than performing replicate analysis of 1 sample. If CV_A_ < CV_I_ to a lesser degree (eg, CV_A_ ≤ 0.5 × CV_I_), as is more likely the case with lower individuality analytes, then replicate measurements of 1 sample with interpretation of the average result (or instituting quality improvement to decrease imprecision) decreases dispersion more than taking multiple samples. It is recommended that CV_A_ should be <0.5 × CV_I_, to decrease variation caused by analytical noise and to make optimal use of dispersion.^21^ Table 2 shows dispersion for commonly measured biochemical and hematology measurands. Example calculations are provided in the Supplemental Appendix. An on‐line dispersion calculator is available from Westgard QC.^21^

**TABLE 2:** Dispersion associated with measurement of common biochemistry and hematology measurands in dogs, cats and horses

	Percent dispersion		
Measurand	Dog	Cat	Horse
Albumin	4	7	6
ALP	22	20	NA
ALT	28	27	NA
AST	19	30	19
GGT	NA	NA	26
CK	64	61	60
Bile acids	NA	255	NA
Total bilirubin	NA	188	43
Cholesterol	23	13	NA
Triglycerides	71	20	49
Creatinine	25	20	17
Urea	27	21	21
Glucose	17	16	16
Total protein	8	7	6
Globulins	NA	9	8
SDMA	33	42	NA
Calcium	6	5	5
Magnesium	NA	10	NA
Phosphate	26	17	25
Sodium	NA	2	3
Chloride	4	2	3
Potassium	5	11	20
TCO_2_	NA	18	NA
RBC	13	11	13
Hemoglobin	13	11	12
Hematocrit	14	11	12
MCV	5	6	2
MCH	4	5	3
MCHC	6	3	3
RDW‐SD	NA	9	NA
RDW‐CV	9	8	8
WBC	35	38	17
Neutrophils	57	50	25
Lymphocytes	88	50	34
Monocytes	104	49	NA
Eosinophils	211	48	NA
Platelets	31	30	38

*Notes*: Percent dispersion for each measurand and species below was calculated using a Z factor of 1.96 (representing 95% bidirectional statistical probability) using the formula $D=\pm Z\times \sqrt{{\mathrm{CV}}_{I}^{2}+{\mathrm{CV}}_{A}^{2}\;}$. CV_I_ and CV_A_ values used for each measurand and species are from the veterinary biological variation database website.^32^

Abbreviations: ALP, alkaline phosphatase; ALT, alanine aminotransferase; AST, aspartate aminotransferase; CK, creatinine kinase; GGT, gamma glutamyl transpeptidase; MCH, mean cell hemoglobin; MCHC, mean cell hemoglobin concentration; MCV, mean cell volume; NA, not available for this species; RBC, red blood cell; RDW‐CV, red blood cell distribution width (coefficient of variation); RDW‐SD, red blood cell distribution width (standard deviation); SDMA, symmetric dimethylarginine; TCO_2_, total carbon dioxide; WBC, white blood cell.

## 3 VETERINARY BV DATABASE

A free, on‐line database summarizing veterinary BV studies is available.^32^ The database provides a list of published veterinary BV studies, including data summaries of these studies. Although all published studies are listed, tables of median results calculated from >1 study only include studies meeting acceptance criteria similar to those used for BV data from humans.^33^ Candidate studies are reviewed by members of the database's advisory committee before inclusion. Over time, the advisory committee recognized that veterinary BV studies are of mixed quality, employing variable study designs and terminology. This led to development of a BV study design and reporting guideline and has stimulated an ongoing effort to improve and standardize study design and nomenclature for veterinary BV studies.^3^ Work to update this database and expand applicability of the information using interactive tools is ongoing.

## 4 CLINICAL DECISION THRESHOLDS AND THEIR LIMITATIONS

In contrast to pRI and iRI, which are statistical estimates representing health, clinical decision thresholds are expert‐based consensus guidelines for disease diagnosis based on clinicopathologic data. Such decision thresholds (eg, for diabetes mellitus diagnosis or treatment) may be different from pRI (eg, for glucose).^34^, ^35^ International Renal Interest Society (IRIS) guidelines for diagnosis and staging of kidney injury or disease are a common example of clinical decision thresholds.^36^ A limitation of such guidelines is that they effectively assume all testing sites can measure with the same degree of precision and accuracy. This is likely not the case, because measurement systems are not necessarily equal, and bias among instruments and test kits using different analytical methods or made by different (or even the same) manufacturer or both are expected. Such bias may be enough to impact patient data interpretation at or near a decision limit.^15^, ^37^ Furthermore, analytical factors (eg, skill of the instrument operator, environmental conditions, differences in instrument age and maintenance) may impact accuracy and precision of the results obtained.^38^ Universal applicability of given decision thresholds is implied by clinical practice guidelines presenting upper and lower decision limits as single numeric values. The IRIS recommendations do acknowledge that analytical methods impact patient clinicopathologic data, but do not provide guidance regarding how variation in analytical methods should be factored into patient data interpretation.^36^

Practical recommendations for applying and developing clinical decision thresholds include:

- Individual veterinarians seeking to monitor serial patient laboratory results over time for purposes of health assessment and clinical staging ideally should choose a single measurement instrument or method and use it consistently, to avoid impact of bias among different instruments or measurement systems.
- Calculating *D* can help veterinarians understand the range of possible values represented by a single patient result. It is particularly useful for results at or near reference limits and medical decision thresholds.
- Patient data should be interpreted as normal or abnormal using statistically valid, instrument‐specific pRI, ideally with knowledge of the relevant measurand's II. Population‐based reference intervals are less diagnostically sensitive for measurands that have high individuality. Calculation and use of RCV or iRI or both can be a useful adjunct to data interpretation using pRI, particularly for measurands with high individuality.
- If a patient has been diagnosed with a defined disease condition, then RCV, iRI, or both can be used for monitoring disease progression (advancing, resolving, or both), response to various treatments (drugs, diet, or other interventions), or both.
- Developers of expert consensus‐based staging and treatment guidelines should recognize the possible impact of analytical performance and BV on recommendations. The impact is likely to be highest for animals having early‐stage disease or results that are at or near clinical decision thresholds. Analytical performance and BV also may impact patient reclassification, when follow‐up evaluation is performed and patients are observed to progress through a series of classifications defined by increasing or decreasing thresholds of laboratory results. In some cases, the impact of analytical imprecision can be mitigated by basing diagnostic and reclassification decisions on average measurements (eg, from multiple specimens or from replicate measurements of 1 specimen). The BV data can be used to estimate the number of replicates needed, the optimal time interval for repeated sampling (see section about estimation of HSP), or both.
- Researchers (eg, of multi‐institutional studies, or of systemic reviews and meta‐analyses) who are analyzing clinicopathologic data generated at >1 testing site should report the measurement methods used and recognize and acknowledge the possible impact of variable analytical performance on study data and conclusions.
- Teams designing research studies or developing consensus guidelines involving clinicopathologic data should include clinical pathologists with expertise in laboratory quality management, reference interval generation and validation, and BV and its applications. Clinical practice guidelines for human medicine often lack input from laboratory specialists, a recognized shortcoming of such documents that leads to omission of information about factors impacting laboratory test results and their interpretation.^39^ Inclusion of laboratory specialists in clinical practice guideline development in human medicine increases the likelihood that considerations relevant for laboratory testing will be addressed.^40^ Developers of veterinary clinical practice guidelines that include decision thresholds for laboratory test interpretation are encouraged to include experienced clinical pathologists in the development team and to review a published checklist of information to include with finalized guideline documents.^40^

## 5 CONCLUSIONS

When interpreting patient clinicopathologic data, veterinarians should be aware that a single measurement result represents a range of possible values, and that a given patient result is impacted not just by the presence or absence of disease, but also by BV and analytical variation. Knowledge of BV and CV_A_ can be used to facilitate patient data interpretation and is particularly useful for interpretation of serial patient data and data at or near reference limits or clinical decision thresholds. Veterinarians should be aware that the various tools (pRI, HSP, RCV, CD, iRI, *D*) used to facilitate patient data interpretation each have benefits and limitations. Inclusion of veterinary clinical pathologists with expertise in quality management and BV on research and veterinary practice guideline development teams can benefit such groups by drawing attention to the various considerations for clinicopathologic data interpretation.

Clinical pathologists with expertise in reference interval generation are potential resources for any investigator or team seeking to develop or validate pRI or interpretation guidelines for a variety of quantitative measurement procedures used in clinical medicine. Many such quantitative metrics (eg, measurements made from imaging studies, force plate metrics in orthopedics, data for monitoring anesthesia) likely have contributions of analytical imprecision and possibly BV to patient results. Interpreting such data will benefit from availability of statistically sound pRI and might be facilitated by BV‐based concepts such as RCV and iRI. Writing interpretation guidelines for such data should incorporate knowledge and discussion of factors that impact measured patient results.

## CONFLICT OF INTEREST DECLARATION

All authors serve on the advisory committee for VetBiologicalVariation.org, a not‐for‐profit veterinary biological variation database. It is anticipated that R. M. Baral and K. P. Freeman will be involved with a commercial application intended to make biological variation‐based tools available to clinicians and clinical pathologists; work on any such application was not begun at the time of this writing.

## OFF‐LABEL ANTIMICROBIAL DECLARATION

Authors declare no off‐label use of antimicrobials.

## INSTITUTIONAL ANIMAL CARE AND USE COMMITTEE (IACUC) OR OTHER APPROVAL DECLARATION

Authors declare no IACUC or other approval was needed.

## HUMAN ETHICS APPROVAL DECLARATION

Authors declare human ethics approval was not needed for this study.

## Supporting information

Additional supporting information may be found online in the Supporting Information section at the end of this article.
