# Supplementary material for: Current and emerging concepts in biological and analytical variation applied in clinical practice
Source: J Vet Intern Med. 2020 Oct 21;34(6):2691–700. doi: 10.1111/jvim.15929 (PMC7694803; doi:10.1111/jvim.15929)
Supplement: Supplementary file 1 — Additional supporting information may be found online in the Supporting Information section at the end of this article. [file JVIM-34-2691-s001.pdf]

SUPPLEMENTAL APPENDIX: EXAMPLE CALCULATIONS A.

Individuality and HSP calculation examples

Three studies have assessed BV of alkaline phosphatase (ALP) in cats.[41-43] All three found CV_G to be approximately 3-fold greater than CV_I, which means variation *between* individuals is substantially greater than variation *within* individuals and that a pRI for feline ALP is likely to be diagnostically insensitive for detecting medically important change *within an individual cat*. High individuality of feline ALP can be shown mathematically by the index of individuality calculation:

$$II = \frac{CV_G}{\sqrt{CV_I^2 + CV_A^2}}$$

To calculate II, median values for CV_G and CV_I from the 3 studies for ALP in cats[32] can be used. In this example, we are using an instrument with CV_A of 1.6%, determined from historical control data.

$$II = \frac{27.8}{\sqrt{(10.2)^2 + (1.6)^2}} = 2.69$$

Index of individuality greater than 1.7 is compatible with high individuality and indicates that iRI or RCV are likely to be more valuable than pRI for patient data interpretation.

To estimate ALP HSP for an individual cat with 95% bidirectional statistical probability, the number of samples needed is determined by the formula:

$$\text{Critical number} = \left(Z \times \frac{\sqrt{CV_I^2 + CV_A^2}}{\text{Dev}} \right)^2$$

To calculate the HSP with 95% bidirectional statistical probability, we use Z = 1.96. For Dev (deviation, referring to allowable deviation around the "true" HSP), we will use 10%. Using median CV_I from the 3 feline ALP BV studies and continuing the example that instrument CV_A is 1.6%,

$$\text{Critical number} = \left(1.96 \times \frac{\sqrt{(10.2)^2 + (1.6)^2}}{10} \right)^2 = 4\ \text{samples}.$$

This means that 4 different blood samples should be measured to estimate the HSP of ALP in an individual cat with a reasonable degree of certainty.

Reference change value, CD, and iRI calculation examples

Using data from 4 years of annual wellness testing, an individual cat's ALP HSP (the mean ALP value calculated from historical wellness data) is 36 U/L. Reference change value for ALP is 31%, as calculated using median feline CV_I for ALP.[32] As above, CV_A is imprecision of the analyzer being used for these assessments, as determined using historical control data. Reference change value 31% means that an increase in ALP activity greater than, or a decrease less than, 31% from 36 U/L is considered medically significant.

Critical difference is determined by multiplying the HSP by the RCV, or in this example, 36 × 0.31 = 11 U/L. A CD of 11 U/L means an increase in ALP activity greater than, or a decrease less than, 11 U/L from 36 U/L is considered medically significant for this cat only.

Individualized reference intervals can be calculated for this cat by using CD and HSP to determine a range. Alkaline phosphatase activities outside this range are considered abnormal for this cat only. In this example, 36 U/L ± 11 U/L = iRI 24 to 47 U/L.

Because ALP in cats is a highly individual analyte (with II approximately 3), iRI for a given individual cat is expected to be considerably narrower than any pRI. Because iRI is calculated from an individual cat's HSP, the resulting range applies only to that individual cat and should not be applied to other cats. In contrast to ALP and other highly individual analytes, iRI of analytes with low individuality (low II) is expected to approximate pRI more closely.

Dispersion calculation examples

Dispersion around a measured result is influenced by imprecision of the analyzer (CV_A) and by the number of samples measured and used to estimate the patient's concentration. In general, (1) for an analyte with a given CV_I, dispersion is expected to increase as assay CV_A increases and (2) for assays with similar CV_A, estimating patient concentration from multiple samples is most beneficial for analytes with larger CV_I.

For example, to calculate dispersion of creatinine concentration in cats, median CV_I (10.2%) calculated from results of 3 prior studies (measuring creatinine in serum and plasma) can be used.[41-43] For creatinine concentration in cats with CV_I 10.2%, dispersion increases approximately 1.2-fold as imprecision changes from 1.3% to 8.0%:

Number of samples	Imprecision (CV_A)	Dispersion
1	1.3%	20.2%
1	2.5%	20.6%
1	3.5%	21.1%
1	4.5%	21.9%
1	5.1%	22.4%
1	8.0%	25.4%

Uncorrected Proofs

Note that CV_A = 8.0% is included here for illustrative purposes—in fact, CV_A = 8.0% would be considered undesirable analytical performance using the commonly applied criterion that CV_A should be less than $0.5 \times CV_I$.

For a creatinine assay in cats with with CV_A of 1.3%, dispersion of results, if 1 sample is measured, is calculated as:

$$\text{Dispersion}\ (\%) = 1.96 \times \sqrt{\left[CV_I^2 + \left(\frac{CV_A^2}{nS}\right)\right]}$$

$$\text{Dispersion}\ (\%) = 1.96 \times \sqrt{10.2^2 + \left(\frac{1.3^2}{1}\right)} = 20\%$$

Figures A1 and A2 illustrate dispersion as applied to International Renal Interest Society (IRIS) stating guidelines for chronic kidney disease (CKD) in cats.[36] A 20% dispersion was calculated around each IRIS creatinine decision limit to illustrate the potential impact of measurement dispersion on patient classification, assuming 1 patient sample is measured (as is often the case in routine clinical practice)

When performing IRIS staging, estimating a patient's true serum or plasma creatinine concentration with greater confidence (higher statistical probability) would require taking >1 serum or plasma sample and using the average creatinine concentration to establish IRIS stage.

For serum or plasma creatinine (CV_I = 10.2%) in cats, there is an approximately 3-fold decrease in dispersion using 10 samples versus 1 sample:

Number of samples	Imprecision (CV_A)	Dispersion
1	1.3%	20.2%
2	1.3%	14.4%
3	1.3%	11.8%
4	1.3%	10.3%
5	1.3%	9.3%
10	1.3%	6.8%

If staging a cat with a true serum or plasma creatinine concentration of 3.4 mg/dL (300 μmol/L) using a creatinine assay with CV_A = 1.3%, the dispersion of measured values around this true concentration is estimated to be 2.7 to 4.1 mg/dL (238 to 362 μmol/L) if 1 sample is measured. In that case, dispersion (predominantly due to creatinine BV, because the assay has good precision) could contribute to misclassification of the cat as IRIS stage 2, when in fact it should be IRIS stage 3. In contrast, if 3 serum or plasma samples are measured and the creatinine concentration averaged, dispersion should be decreased, and the range of possible values closer to 3.0 to 3.8 mg/dL (265-336 μmol/L), with a higher probability of accurate staging. Of course, patient stress and owner expense of taking and measuring >1 sample must be weighed against confidence in the result(s). This issue may be less important for individual cats, but it should be considered during the design of scientific studies of CKD where groups of cats

Uncorrected Proofs

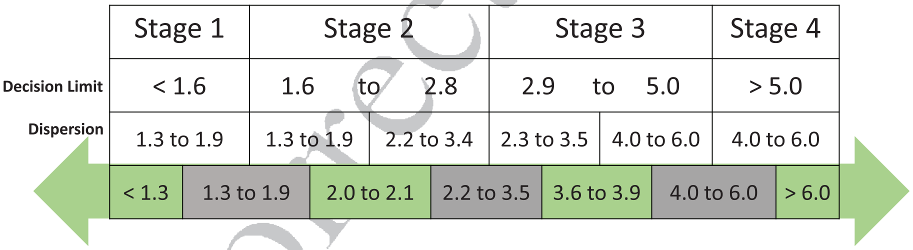

FIGURE A1 Serum or plasma creatinine concentration dispersion (mg/dL) and IRIS CKD decision thresholds in cats. Green zones indicate serum or plasma creatinine concentrations for which confidence in correct patient classification is highest (at least 95% statistical probability). Gray zones indicate serum or plasma creatinine concentrations for which confidence in correct patient classification is less, because dispersion of creatinine measurement means overlap of results for patients at or near decision thresholds is possible

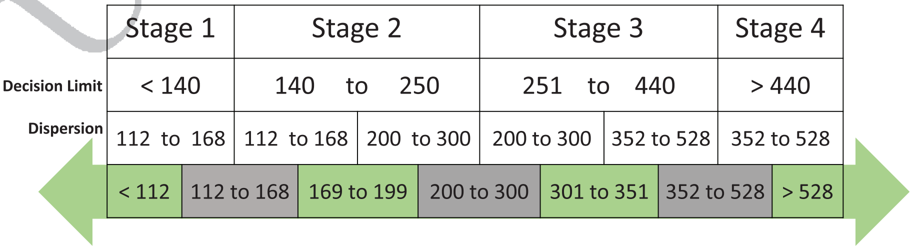

FIGURE A2 Serum or plasma creatinine concentration dispersion (mmol/L) and IRIS CKD decision thresholds in cats. Green zones indicate serum or plasma creatinine concentrations for which confidence in correct patient classification is highest (at least 95% statistical probability). Gray zones indicate serum or plasma creatinine concentrations for which confidence in correct patient classification is less, because dispersion of creatinine measurement means overlap of results for patients at or near decision thresholds is possible

with different disease stages are compared. The IRIS staging guidelines currently do not include a recommendation for how many samples to measure when making decisions about IRIS classification, and achieving consensus on this point would require discussion among internists and clinical pathologists versed in BV and analytical performance of the relevant assays.

Uncorrected Proofs
